# Host Genetics Background Influence in the Intragastric *Trypanosoma cruzi* Infection

**DOI:** 10.3389/fimmu.2020.566476

**Published:** 2020-11-24

**Authors:** Carolina Salles Domingues, Flávia de Oliveira Cardoso, Daiana de Jesus Hardoim, Marcelo Pelajo-Machado, Alvaro Luiz Bertho, Kátia da Silva Calabrese

**Affiliations:** ^1^ Laboratório de Imunomodulação e Protozoologia, Instituto Oswaldo Cruz, Fundação Oswaldo Cruz, Rio de Janeiro, Brazil; ^2^ Laboratório de Patologia, Instituto Oswaldo Cruz, Fundação Oswaldo Cruz, Rio de Janeiro, Brazil; ^3^ Laboratório de Imunoparasitologia, Instituto Oswaldo Cruz, Fundação Oswaldo Cruz, Rio de Janeiro, Brazil; ^4^ Plataforma de Citometria de Fluxo, Instituto Oswaldo Cruz, Fundação Oswaldo Cruz, Rio de Janeiro, Brazil

**Keywords:** *Trypanosoma cruzi*, inbred mice, intragastric infection, host genetic background, proinflammatory cytokines, CD8^+^ lymphocytes

## Abstract

**Background:**

Considering the complexity of the factors involved in the immunopathology of Chagas disease, which influence the Chagas’ disease pathogenesis, anti-*T. cruzi* immune response, and chemotherapy outcome, further studies are needed to improve our understanding about these relationships. On this way, in this article we analyzed the host genetic influence on hematological, histopathological and immunological aspects after *T. cruzi* infection.

**Methods:**

BALB/c and A mice were intragastrically infected with *T. cruzi* SC2005 strain, isolated from a patient of an outbreak of Chagas disease. Parameters such as parasite load, survival rates, cytokines production, macrophages, T and B cell frequencies, and histopathology analysis were carried out.

**Results:**

BALB/c mice presented higher parasitemia and mortality rates than A mice. Both mouse lineages exhibited hematological alterations suggestive of microcytic hypochromic anemia and histopathological alterations in stomach, heart and liver. The increase of CD8^+^ T cells, in heart, liver and blood, and the increase of CD19^+^ B cells, in liver, associated with a high level of proinflammatory cytokines (IL-6, TNF-α, IFN-γ), confer a resistance profile to the host. Although BALB/c animals exhibited the same findings observed in A mice, the response to infection occurred later, after a considerable parasitemia increase. By developing an early response to the infection, A mice were found to be less susceptible to *T. cruzi* SC2005 infection.

**Conclusions:**

Host genetics background shaping the response to infection. The early development of a cytotoxic cellular response profile with the production of proinflammatory cytokines is important to lead a less severe manifestation of Chagas disease.

## Introduction

Chagas’ disease is caused by *Trypanosoma cruzi* and it is estimated that there are about 6 to 7 million people infected in the worldwide (1). This parasite presents a great genetic variation and these differences are responsible for a remarkable diversity of isolates, which are classified in seven Discrete Typing Units (DTUs), TcI–TcVI, and Tcbat (2–5).

Since it was described in 1909, Chagas’ disease has presented a number of different epidemiological profiles. The evolution of these epidemiological scenarios is due to the various forms of *T. cruzi* transmission, as well as the development of the means used for its prevention. Despite of the progress made to control of infection and the continuous support of a number of organizations, Chagas’ disease still affects low-income populations, although has been expanded to North America, Europe, Australia and Japan by migration of million people from endemic countries (6–8). The varied routes of the disease transmission continue to occur, ensuring the continued existence of this zoonosis. However, oral transmission has been showing the main route of infection in the last decades (2, 9, 10).

Outbreaks of acute Chagas disease caused by oral infection have been reported frequently in the Amazon Basin [(11), reviewed by (9)]. Most cases of Chagas disease acquired by oral route were due to TcI isolates, with rare cases involving TcIII and TcIV (12–14). Outbreaks associated with TcII have been reported in southern Brazil (3). Clinical manifestations of Chagas’ disease developed after oral infection are more serious than those acquired through the vector pathway. They include acute myocarditis with heart failure, prolonged fever and in some cases, meningoencephalitis (15–17). The disease outcomes depend on several factors such as: both parasite and host genetics, immune response, the route of infection, evolutive forms of the parasite, mixed infections, and cultural and geographical factors (3, 18–22).

Lewis et al. (22) showed the influence of transmission route, evolutive form of parasite and inoculum in *T. cruzi* infection. In this study, 92% of mice infected with 10^4^ metacyclic (MT) and 100% of mice infected by intraperitoneal route, with bloodstream trypomastigotes (BT) of *T. cruzi* clone CL Brener exhibited a disseminated infection, with higher parasite loads in the spleen, gastrointestinal tract and adipose-rich tissues. In mice infected intragastrically with 10^4^ MTs, about 25% of mice were infected; when the inoculum was increased to 10^5^, the infectivity was boosted to 67% and parasites were only detected in the stomach. The mice were not infected with BTs by intragastric route. When oral infection via the oral cavity was realized using 10^4^ BT trypomastigotes, 36% of mice were infected, while infection with 10^4^ MTs was not transmissible via the oral cavity. When the inoculum was increased to 10^5^, infectivity was boosted to 100% in MT infected mice. Parasite load was undetectable in any organ/tissue from animals infected by this route.

A study of our group, using Swiss mice infected intragastrically (IG) or intraperitoneally (IP) by *T. cruzi* SC2005 strain, derived from an outbreak of oral Chagas disease, showed also the influence of route transmission on *T. cruzi* infection. All IP-injected animals showed parasitemia, while just 36% of IG infected mice showed the presence of the parasite in the blood. The parasite load in the blood and mortality rate is greater in mice IP-infected, than in IG-infected mice. The pattern of tissue colonization was the same in both groups of infected mice, regardless of the route of infection, showed great damage in the heart, with the presence of large lymphocytic inflammatory infiltrates (23).

The immune response is another factor that influences the outcome of disease and is determined by the interaction between both parasite and host genetics. Several inbred mouse strains when infected with *T. cruzi* exhibit different profiles of response to infection. Differences in mortality rates, cytokine production, inflammatory infiltrates and parasite load are observed, determining different degrees of susceptibility in the hosts (24–26). CBA mice infected with *T. cruzi* SC2005 strain showed high mortality and parasitemia rates and a Th2/Th17 profile of cytokine production, while C57BL/10 showed lower rates of parasitemia and mortality and a Th1/Th2/Th17 cytokine profile (25). Silva et al. (24) studying the infection by *T. cruzi* Y in several inbred mouse strains (A/J, BALB/c, C3H/HePas, C57BL/6, and DBA mice) also showed differences in mortality and parasitemia rates. A/J mice were the most susceptible to infection, showing the highest rates of parasitemia, mortality, number of inflammatory cells in the liver and number of amastigotes per cell, while the C57BL/6 was the strain less susceptible to infection. In another study, Ferreira et al. (26) observed that BALB/c mice infected by G and CL strains of *T. cruzi* better regulate the immune response than C57BL/6 mice, since BALB/c animals initiated the cytokine secretion earlier. These works demonstrate that factors related to the host genetics are important to control the infection

Studies have been illustrated that the genetic background of the host influence the *T. cruzi* tissue tropism and Chagas disease presentations. Andrade et al. (27) using BALB/c, DBA/2, and C57BL/6 infected by TcI and TcII strains showed that a differential tissue distribution was linked with the MHC variability of the host and no with parasite genetic. Since, BALB/c and DBA/2 mice have the same H-2 haplotype (d) and C57BL/6 mice have H-2 (b) (27, 28). Researches on polymorphisms of some MHC alleles in Chagas’ disease patients from Brazil (29); Venezuela (30) and Mexico (31) showed that there is an association between some MHC alleles and disease presentations (32, 33).

Considering the complexity of the factors involved in the immunopathology of Chagas disease, which influence the Chagas’ disease pathogenesis, anti-*T. cruzi* immune response and chemotherapy outcome, there is the need for further studies to improve our understanding about these relationships. On this way, the present study used *T. cruzi* SC2005 strain (TcII), isolated from a human case of an oral outbreak of Chagas’ disease in the south region of Brazil (34), to infect inbred mice from different genetic backgrounds and to conduct a comparative analysis of the immunopathological, histopathological and hematological profiles, using parameters such as of parasite load, survival rates, cytokines production, macrophages, T and B cell populations frequencies and histopathology. In this study, we observed that although the infection was disseminated in A and BALB/c mice, A animals were less susceptible to *T. cruzi* SC2005 infection. This lineage developed an early cytotoxic cellular profile, before the increase in parasitemia, leading to a less severe manifestation of Chagas disease.

## Material and Methods

### Ethics Statement

Experiments were conducted following the guidelines for experimental procedures of the National Council for the Control of Animal Experimentation and approved by the Ethics Committee for Animal Research of the Fundação Oswaldo Cruz (CEUA-FIOCRUZ), license LW 42/14.

### Animals

Four to 6 weeks old female mice of the A and BALB/c inbred strains were used in the experiments, provided by Instituto de Ciência e Tecnologia em Biomodelos (FIOCRUZ) and housed under pathogen-free conditions, controlled temperature and food and water *ad libitum*.

### Parasites

*Trypanosoma cruzi* SC2005, belonging to DTU Tc II, was isolated from the peripheral blood of a patient in the acute phase of Chagas disease, acquired orally during an outbreak in Santa Catarina, Brazil, in 2005 (34, 35).

Epimastigote forms of *T. cruzi* SC2005 strain were maintained in LIT (Liver Infusion, Triptose) (AGM) culture medium at 28°C for 21 days, when typically 70%–90% of the parasites had differentiated (36). Metacyclic trypomastigotes were quantified in a Neubauer hemocytometer prior to infection (37).

### Experimental Design

A and BALB/c mice were organized into four experimental groups, as follows: Group 1 (G1; n=70): A mice intragastrically infected by 10^7^ metacyclic trypomastigote forms of *T. cruzi* SC2005 strain/0.3 ml of LIT medium using a gavage needle; Group 2 (G2; n=70) BALB/c mice, infected under the same experimental conditions as G1; and Group 3 (G3; n=50) and Group 4 (G4; n=50)—control groups, each one composed by uninfected A and BALB/c mice, respectively. In the experimental groups, BALB/c and A mice were submitted to 4 h of fasting prior to intragastric injection. Six animals from each group were euthanized prior to the removal of the blood, esophagus, stomach, gut, heart and liver. Organs were obtained at 7, 14, 21, and 40 days post-infection (dpi). Two independent experiments were performed.

### Parasitemia and Mortality

To compare the parasitemia and mortality caused by *T. cruzi* SC2005 intragastric infection between A and BALB/c strains, twenty mice from each group (G1 and G2) were monitored. The parasitemia was determined daily from 5 to 40 dpi. Briefly, 5 µl of blood from each animal’s tail vein were collected and placed between slide and cover slip (22 x 22 mm). The number of parasites/ml of blood was estimated by counting 50 microscopic fields in a 400X magnification as described by Pizzi and Prager (38).

The mortality was followed daily until the 50^th^ day after infection. The mortality rate was estimated according to the survival curve generated by the GraphPad Prism 6 program.

### DNA Extraction

To quantify the parasite load on the stomach (site of inoculation) and heart throughout the infection in both infected mouse strains, immediately after euthanasia, fragments of both organs (six animals/group) were removed (approximately 100 mg), immediately frozen and stored at—70 °C. After, fragments were digested in 500 µl of lysis buffer (50 mM Tris, 10 mM NaCL, 5 mM EDTA, 0,5% SDS) containing proteinase K (20 mg/ml). DNA was extracted following a standard phenol/chloroform protocol (39).

DNA concentrations and purity were determined by reading A260 and A280 on a NanoDrop 2000c spectrophotometer (Thermo Fisher Scientific, Wilmington, DE, USA).

### qPCR Primer Design and Assay Conditions

All primers were designed to be used in a SYBR Green qPCR assay. Primers Cruzi 1 and Cruzi 2 targeting *T. cruzi* genomic DNA sequence (166 bp) and *Actb—actin, beta* (β-actin) mouse gene (138 bp) were synthesized as previously reported (40, 41) (**Table 1**). All primers were manufactured by Gene Link (Hawthorne, NY). The *Actb* reference gene was used as a positive control to monitor DNA integrity, the presence of potential inhibitors of PCR or variation in DNA yield.

**Table 1:** Primers used for real-time PCR.

Target	Primer sequence^a^		Sequence source
	Forward	Reverse	
**β-actin**	AGAGGGAAATCGTGCGTGAC	CAATAGTGATGACCTGGCCGT	X03672/V01217
**Cruzi 1**	ASTCGGCTGATCGTTTTCGA	–	AY520036
**Cruzi 2**	–	AATTCCTCCAAGCAGCGGATA	AY520036

^a^S, C/G.

All reactions were performed using StepOnePlus Real-Time PCR System (Applied Biosystems). The reaction mixtures contained Power SYBR Green PCR Master Mix 2X (Applied Biosystems), 300 nM of Cruzi1/Cruzi2 or 100 nM of β-actin primers and 25 ng of DNA template in a final volume of 20 µl. PCR conditions were as follows: hold at 95°C for 10 min, followed by 40 temperature cycles of 95 °C for 15 s and 58 °C for 1 min. Standard curves were generated from 10-fold serial dilutions of axenic epimastigotes *T. cruzi* DNA (100 ng – 1 pg). PCR reactions were performed as triplicates for each sample.

A melt curve analysis was performed on all reactions. The quality parameters of the standard curves were analyzed with the StepOne software v2.2.2 (Applied Biosystems).

### Histopathology

Histopathological analysis was used to describe the alterations found throughout the infection, in the different organs of infected mice. Following euthanasia, fragments of the esophagus, stomach, gut, heart, and liver of six animals/group were fixed in 4% paraformaldehyde in phosphate buffered saline (PBS), pH 7.45, at 4 °C, for 72 h, cleaved and routinely processed paraffin-embedded. Tissue sections (5µm) were stained with Hematoxylin-Eosin (HE) (Sigma-Aldrich, Sant Louis, USA) technique. The presence of inflammatory infiltrates was classified as: (-) without infiltrates, (+) very mild lesion areas, (++) mild lesion areas, (+++) moderate areas of infiltrates, (+++) severe areas of infiltrates, (++++) very severe areas of infiltrates, follow described by Barreto-de-Albuquerque et al. (42). Tissues were analyzed and photographed under light microscopy (Zeiss, Axioplan 2, with Axiovision LE64 photomicrograph equipment).

### Hematological Analysis

Hematological changes occurred after *T. cruzi* SC2005 intragastric infection were determined by the complete blood count (CBC) of six animals/group, in each euthanasia point. The blood was collected by cardiac puncture, placed in EDTA tubes, and sent to the Laborlife clinical laboratory (RJ, Brazil). The blood parameters evaluated were: Red Blood Cell (RBC), Hemoglobin (Hgb), Hematocrit (Hct), Mean Corpuscular Volume (MCV), Mean Corpuscular Hemoglobin (MCH), Mean Corpuscular Hemoglobin Concentration (MCHC), White Blood Cell (WBC) and Platelet (PLT). The CBC was analyzed in an automatic cell counter (Beckman Coulter, Brea, CA).

### Cytokine Analysis

Cytokine profile induced by the infection was performed through BD Cytometric Bead Array (CBA) Mouse Th1/Th2/Th17 Cytokine Kit (cat. 560485; BD Biosciences, San Jose, CA) using flow cytometry. Briefly, the detection of TNF-α, IFN-γ, IL-10, IL-6, IL-17A, IL-4, and IL-2 in the serum of animals, was realized using 50 µl of each sample, 50 µl of capture beads and 50 µl of mouse Th1/Th2/Th17 PE detection reagent following CBA-kit instruction guide. Samples were acquired in the BD FACSCalibur flow cytometer (BD Biosciences). The data were analyzed with FCAP Array software (BD Biosciences).

### Obtaining of Mononuclear Cells to Flow Cytometry

In the determined euthanasia points, the blood of each animal was collected by cardiac puncture using citrated saline (0.87% NaCl, 3.8% Na_3_C_6_H_5_O_7_), diluted in the same volume of complete RPMI medium—RPMI 1640 medium (Sigma-Aldrich, St. Louis, MO), supplemented with 10% fetal bovine serum (FBS) (CultiLab, Campinas, Brazil); 200 mM L-glutamine; 100 U/ml penicillin; and 10 μg/ml streptomycin (Sigma-Aldrich, St. Louis, MO), being then added to a Ficoll-Hypaque (Histopaque 1077; Sigma-Aldrich) sedimentation gradient. After centrifugation at 1030 x g for 20 min at 21 C, without brake, the mononuclear cells (MCs) ring was collected.

A lobe of the liver was macerated in 4 ml of complete RPMI medium until complete disruption using a glass tissue homogenizer (Corning E.U.A.), being kept on ice throughout the process.

Cardiac cells were obtained after organ perfusion with PBS (pH 7.2), subsequently cut into small fragments and subjected to four cycles of dissociation using 0.2% type II collagenase in RPMI medium without FBS at 37°C, stirring for 30 min. The supernatant obtained after each dissociation cycle was collected in a single tube and kept on ice.

After obtaining, cells of blood, liver and heart were washed twice (with centrifugation at 720 x g, 4°C, 5 min) in PBS-BSA-(Bovine Serum Albumin) with 10% FBS and resuspended with 3 ml of ACK (Ammonium-Chloride-Potassium) Lysing Buffer, to lyse of red blood cells, incubated for 5 min at RT and washed again. After that, cells were incubated in PBS-BSA with 10% FHS (Fetal Horse Serum) for 30 min, washed again, resuspended with complete RPMI medium and adjusted to 1x10^6^/well.

### Flow Cytometry

Flow cytometry was used to quantify the subpopulations of inflammatory cells present in the whole blood (T and B cells), heart and liver (macrophages, T and B cells) of six mice from each experimental group after *T. cruzi* SC2005 intragastric infection.

After each obtaining protocol described above, cells were submitted to flow cytometry staining protocol, consisted by the following monoclonal-antibody panel: anti-CD3-PC7 (Cat 553064; BD Biosciences—maximum emission (max-em): 785 nm) diluted 1:40; anti-CD4-APC-H7 (Cat 560181; BD Biosciences—max-em: 785 nm) diluted 1:80; anti-CD8-BB515 (Cat. 564422; BD Biosciences—max-em: 515 nm) diluted 1:160; anti-CD19-APC (Cat MCA 1439; Serotec—max-em: 661 nm) diluted 1:20 and anti-F4/80-Alexa Fluor® 700 (Cat 123130; Biolegend—max-em: 719 nm) diluted 1:50, in PBS-BSA-FHS. Cells were incubated for 20 min in the dark at room temperature. After washing with PBS, to remove unbound antibodies, the cells were fixed in 1% paraformaldehyde for 30 min in the dark at 4°C, washed and stored at 4°C in the dark until acquisition in flow cytometry. At least 20,000 events from each sample were acquired through CytoFlex flow cytometer (Beckman Coulter). Single stained controls were used to set compensation parameters, while unstained cells were used to set analysis regions. After acquisition, flow cytometric analysis to evaluate the frequencies of CD8^+^T, CD4^+^T, CD4^+^/CD8^+^ T, CD19^+^B, and macrophagic cells were performed using CytoExpert Software (Beckman Coulter). A gate strategy was performed as follows (**Figure S1**): to exclude cell aggregates from analyses, cells were gated on **Singlets** region in FSC-A *vs* FSC-H dot-plot (**Figure S1A**); from **Singlets** gate an FSC-A *vs* Side-Scatter-Area (SSC-A) dot plot was created and analyses region (mononuclear cells) was defined to encompass mononuclear cells and exclude dead cells from analyses (**Figure S1B**); from **Mono** gate, CD4^+^ and CD8^+^ T lymphocytes were determined by CD3 *vs* CD4 and CD3 *vs* CD8 dot plots, respectively (**Figures S1C, D**), CD19+B cells by CD3 *vs* CD19 dot plot (**Figure S1E**) and F4/80^+^ macrophages by CD3 *vs* F4/80 dot plot (**Figure S1F**). CD4^+^/CD8^+^ double positive T cells was determined by plotting CD8 *vs* CD4 gated on CD3^+^ (**Figure S1G**).

### Statistical Analysis

Data were expressed by mean ± standard error of the mean (SEM) and analyzed statistically by Two-way Anova and Tukey´s multiple comparison post-test. The analyses were performed using the GraphPhad Prism 6 software. Differences were considered significant when p<0.05.

## Results

### Parasitemia and Mortality

**Figure 1:**
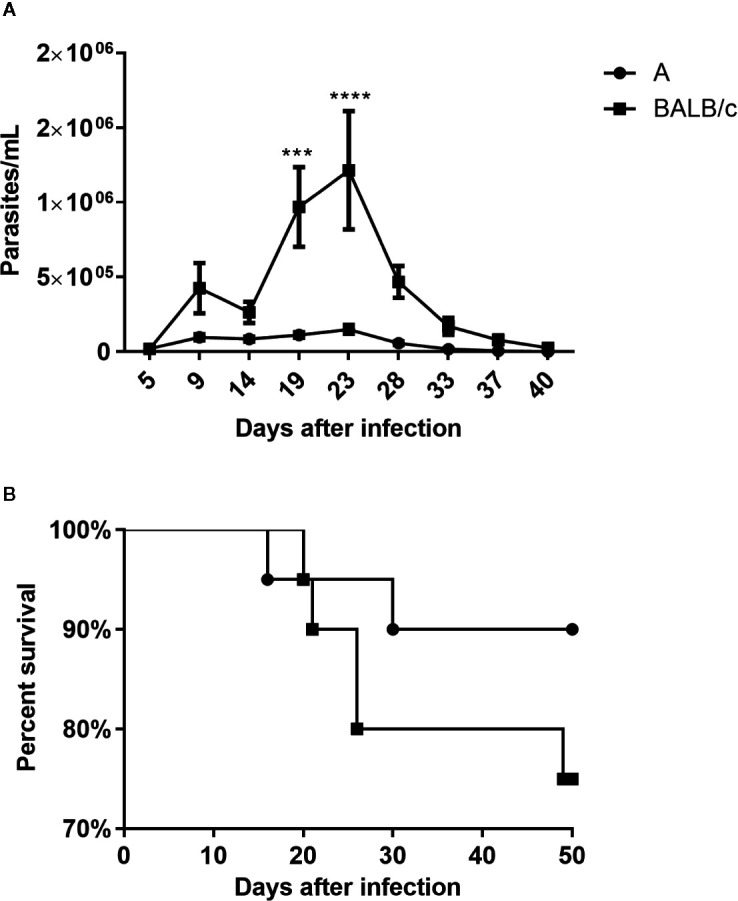
Parasitemia and mortality—A Number of parasites/ml **(A)** and percent survival **(B)** of A and BALB/c mice intragastrically infected by 10^7^ metacyclic trypomastigote forms of *T. cruzi* SC2005 strain. Data represent mean ± SEM of two independent experiments with 20 animals/group. Statistical analyses between groups were performed using Two-way Anova and Tukey multiple comparison test. Results were considered significant with P < 0.05 (***P < 0.001; ****P < 0.0001).

All animals of both infected mouse strains presented detectable parasitemia. BALB/c mice showed parasitemia levels significantly higher than A mice, with peaks at 9 and 23 dpi (**Figure 1A**).

Although A mice presented an earlier mortality (16 dpi) its survival percentage was higher, at around 90%, than observed in the BALB/c mice (75%) at 50 dpi (**Figure 1B**).

### Parasite Load

**Figure 2:**
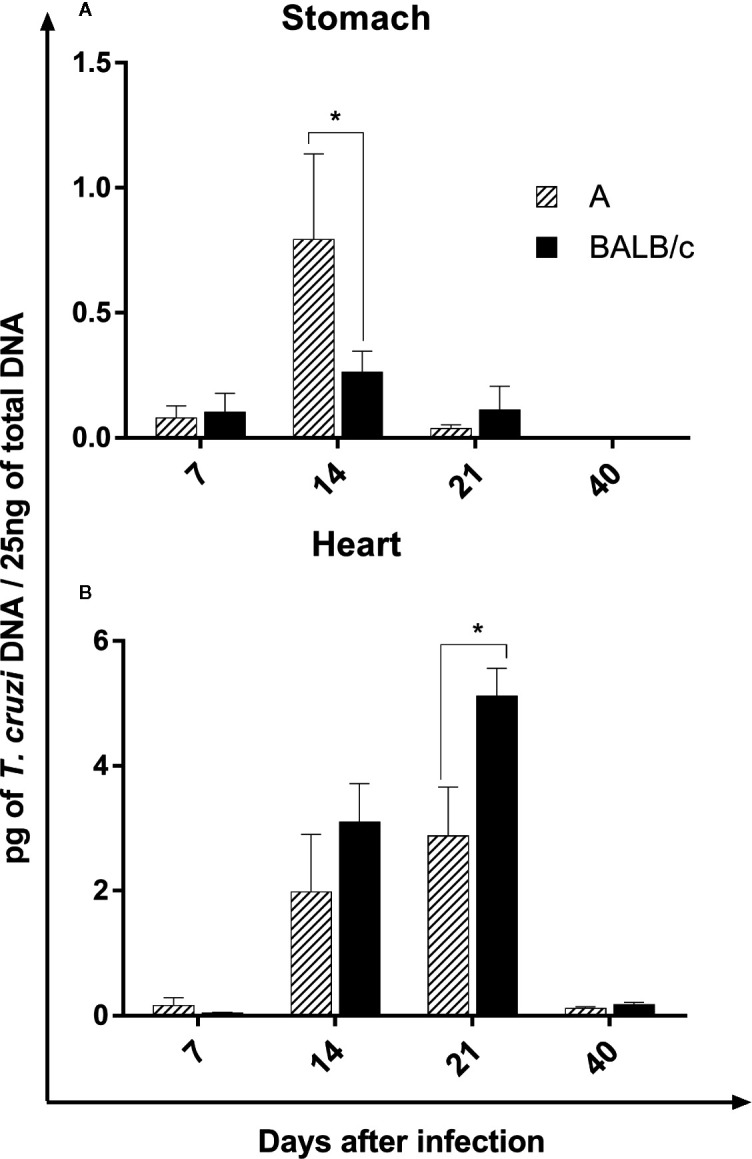
Parasite Load—Quantification of parasites by qPCR in the stomach **(A)** and heart **(B)** of A and BALB/c mice intragastrically infected by 10^7^ metacyclic trypomastigote forms of *T. cruzi* SC2005 strain. Data represent mean ± SEM of two independent experiments with six animals assayed in triplicate. Statistical analyses between groups were performed using Two-way Anova and Tukey multiple comparison test. Results were considered significant with *P < 0.05.

The parasite load in the stomach was low in both infected mouse strains. However, both mouse strains showed an increase in the parasite load at 14 dpi, which was significantly higher in A mice than in BALB/c mice, reducing in later times (**Figure 2A**).

In the heart, the parasite load increase at 14 and 21 dpi in both infected lineages. Contrary to observed in the stomach, BALB/c mice showed a significantly higher parasite load when compared with A mice, mainly at 21 dpi, however, at 40 days after infection, the parasite load drastically reduce in both strains (**Figure 2B**).

The parasite load in both organs was significantly lower at 40 dpi, when compared to previous moments. In addition, the heart showed a higher quantity of *T. cruzi* DNA in relation to the stomach in both mouse strains (**Figures 2A, B** **)**.

### Histopathology

Histopathological analysis of both mouse strains showed similar alterations, with a few modifications in esophagus, stomach, heart and liver throughout the infection. No histopathological alterations were observed in the gut of both infected mice at any time. Neither no alterations were showed in esophagus of infected BALB/c animals during the study. The intensity of inflammatory infiltrates in studied organs was similar in both mouse infected strains, except in esophagus of BALB/c mice, which showed no inflammatory infiltrate. A slight decrease in the inflammatory infiltrate intensity was observed in the stomach (after 21 dpi) and heart (after 14 dpi) of A infected mice (**Table 2**).

**Table 2:** Semiquantitative analysis of the inflammatory infiltrates in different organs after *T. cruzi* SC2005 infection.

Organs	dpi	Mouse strains	
		BALB/c	A
**Esophagus**	7	–	–
	14	–	++
	21	–	+++
	40	–	++
**Stomach**	7	++	++
	14	+++	+++
	21	++++	+++
	40	+++	++
**Gut**	7	–	–
	14	–	–
	21	–	–
	40	–	–
**Heart**	7	++	++
	14	++++	+++
	21	+++++	++++
	40	++++	++++
**Liver**	7	++	+
	14	++	++
	21	++	++
	40	++	++

dpi, days post infection; (-) without infiltrates; (+) very mild lesions areas; (++) mild areas of infiltrates; (+++) moderate areas of infiltrates; (++++) severe areas of infiltrates; (+++++) very severe areas of infiltrates.

Seven days post infection, A infected mice showed a small area of diffuse inflammatory infiltrates in the stomach and heart. In the mucosa, submucosa and muscularis layers of the stomach, mild areas with lymphomonocytic infiltrates were observed (++). The heart showed mild infiltrates in the atrium (++) composed essentially of lymphocytes (**Table 2**). BALB/c mice showed histopathological changes in the stomach, heart and liver. Stomach showed mild heterogeneous inflammatory infiltrate (++) in the mucous layer. In the heart, as also observed in the infected A mice, BALB/c mice showed lymphocytic infiltrates in the atrium (++). The liver presented focal and mild inflammatory infiltrate (++), close to the liver portal space (**Table 2**).

**Figure 3:**
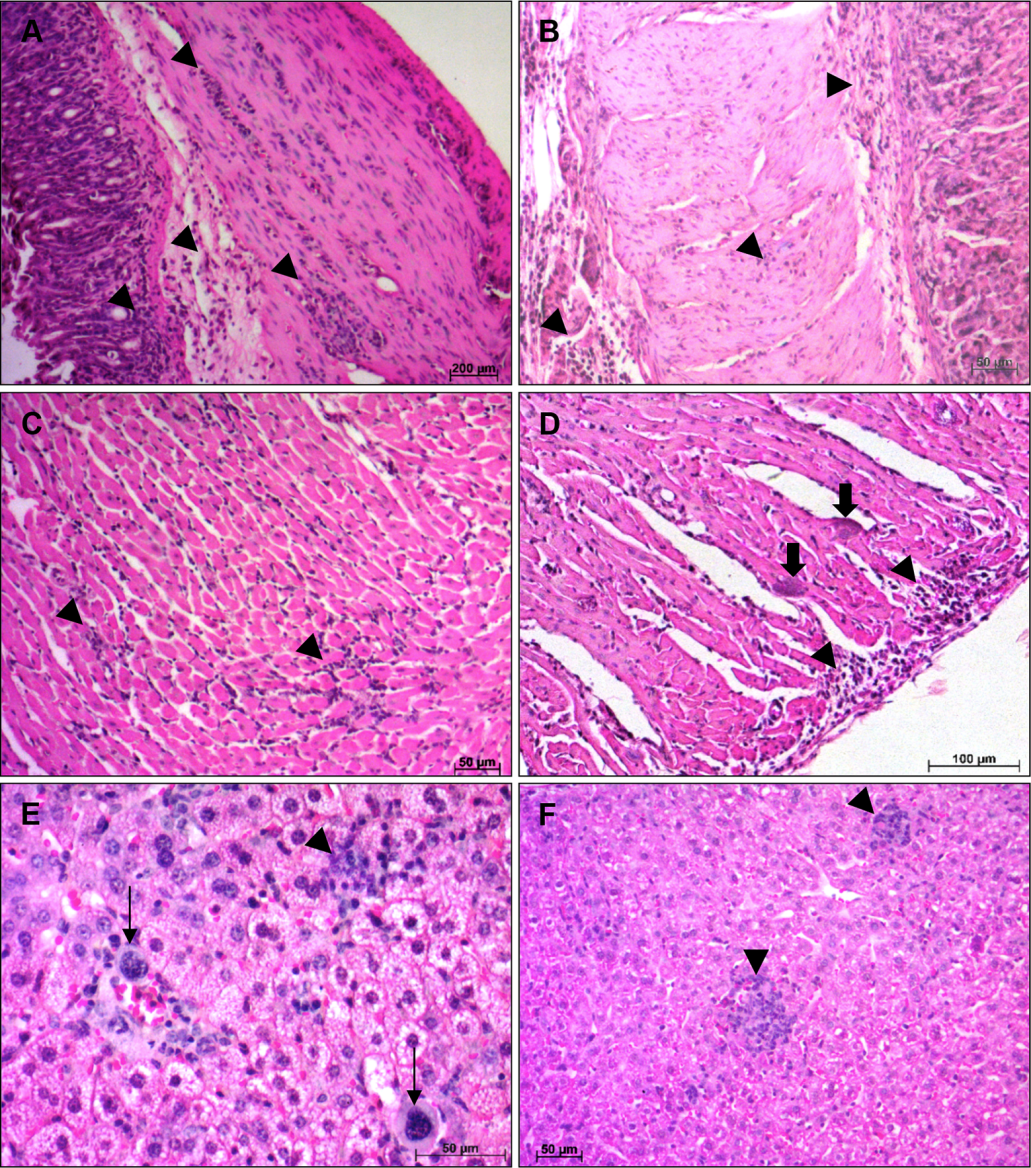
Histological alterations at 14 days post infection—Histopathological alterations in stomach, heart and liver of A and BALB/c mice intragastrically infected by 10^7^ metacyclic trypomastigote forms of *T. cruzi* SC2005 strain, at 14 days post infection. **(A)** Lymphomonocytic inflamatory infiltrate (arrowhead), in the stomach of A mice, from mucosa until muscular layer; **(B)** Stomach of BALB/c mice showing inflammatory infiltrates (arrowhead) from mucosa until serosa layer; **(C)** Heart ventricle of A mice with diffuse inflammatory infiltrate (arrowhead); **(D)** Parasites nests (thick arrow) in heart ventricle and diffuse inflammatory infiltrate (arrowhead) in the pericardium of BALB/c mice; **(E)** Lymphocytes, macrophages and megakaryocytes (thin arrow) in the liver of A mice. **(F)** Focal inflammatory infiltrate (arrowhead) in liver of BALB/c mice. Hematoxylin-Eosin (HE) staining method. n = 6 mice/group (three different sections from each mouse).

As the infection progressed, both mouse strains showed an increase, in the intensity and extent, of inflammatory infiltrates in the different analyzed organs. At 14 dpi, infected A mice exhibited diffuse inflammatory infiltrates of different intensities and distribution in esophagus, stomach and heart. The presence of sparse inflammatory cells was observed in the muscular layers of the esophagus (++). The stomach showed a great circulation of inflammatory cells, with moderate areas of diffuse and intense inflammatory infiltrates (+++), essentially composed by lymphocytes, extended from mucosa to muscular layer, however the inflammatory cells were in greater quantity in muscular layer, which was more affected. (**Figure 3A**). In the heart, the atrium was more inflamed than the ventricle, with an increase in the number of inflammatory cells circulation (+++) and the presence of few *T. cruzi* amastigotes nests (**Figure 3C**). The liver presented small foci of inflammatory cells (++), essentially lymphocytes and macrophages, as well as the presence of megakaryocytes and immature hematopoietic cells (**Figure 3E**). Infected BALB/c mice, showed histopathological changes in stomach, heart and liver. The stomach present moderate areas of diffuse inflammatory infiltrates extended from lamina propria to serosa layer (+++), in the muscular layer biggest areas of inflammatory infiltrates were observed (**Figure 3B**). A moderate inflammatory infiltrate, predominantly composed by lymphoid cells, was observed in the heart, with the atrium being the most affected region (+++). In the ventricle, intense pericarditis, and numerous *T. cruzi* nests were noted (**Figure 3D**). The liver showed moderate and focal inflammatory infiltrates, essentially mononuclear, distributed regularly throughout the organ (++) (**Figure 3F**). Granulocytes, plasma cells and megakaryocytes were noted circulating in the tissue.

**Figure 4:**
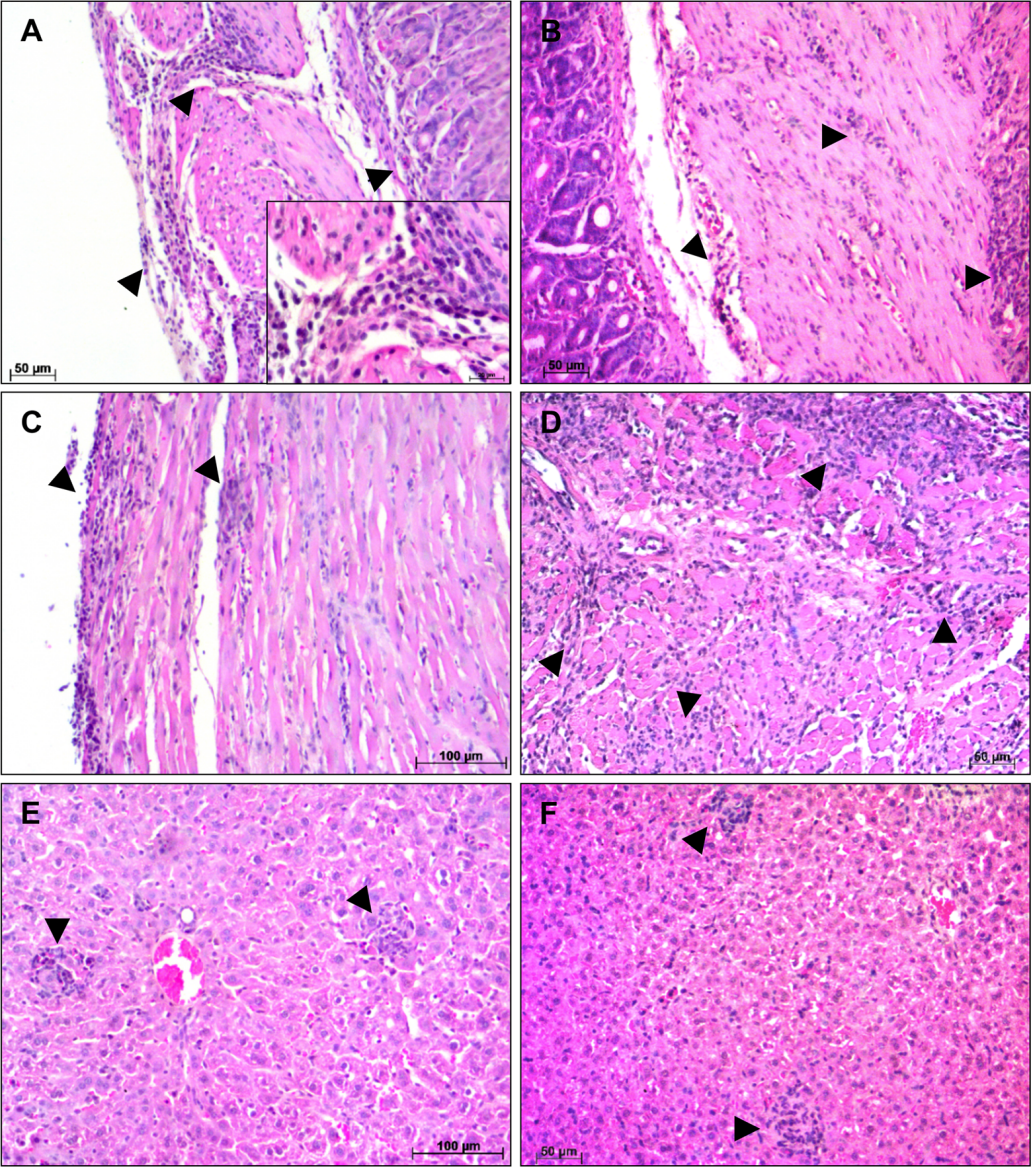
Histological alterations at 21 days post infection—Histopathological alterations in stomach, heart and liver of A and BALB/c mice intragastrically infected by 10^7^ metacyclic trypomastigote forms of *T. cruzi* SC2005 strain, at 21 days post infection. **(A)** Stomach of A mice showing a diffuse inflammatory infiltrate (arrowhead), with greater involvement of the muscular layer; **(B)** Lymphomonocytic inflammatory infiltrate (arrowhead) in the stomach of BALB/c mice causing tissue damage in muscular layer; **(C)** Endocardium of the A mice heart showing diffuse inflammatory infiltrate (arrowhead); **(D)** Heart atrium of BALB/c mice with intense inflammatory infiltrate (arrowhead); **(E)** Focal inflammatory infiltrate (arrowhead) in the liver of A mice; **(F)** Focal inflammatory infiltrate in the liver of BALB/c mice. Hematoxylin-Eosin (HE) staining method. n = 6 mice/group (three different sections from each mouse).

At 21 dpi, A infected mice showed areas of moderate diffuse inflammatory infiltrates in the muscular layer of the esophagus (+++). In the stomach, the mucosa, submucosa and muscular layers remained inflamed, but at this point of infection, the tissue damage between the muscle fibers was greater (++++) (**Figure 4A**). The heart showed diffuse inflammatory infiltrates, composed by lymphocytes and plasma cells, between the muscle fibers. The atrium was still more affected than ventricle, with the pericardium and endocardium being the most inflamed regions (++++) (**Figure 4C**). Although the number of inflammatory cells is visually smalls in the ventricle, only in this region were observed *T. cruzi* nests. Examination of liver showed mild areas of focal and heterogeneous inflammatory infiltrate (++), with the presence of immature hematopoietic cells, megakaryocytes and mitosis figures (**Figure 4E**). Infected BALB/c mice stomachs showed a greater tissue damage, with intense and diffuse inflammatory infiltrates (++++). The presence of edema and the separation of fibers from the muscular layers was noticed (**Figure 4B**). At that moment of infection, heart, as well as the stomach, showed more severe histopathological changes. Severe areas of diffuse inflammatory infiltrates were observed in the atrium and ventricle, leading to an injury of muscle fibers, with necrosis and edema (+++++) (**Figure 4D**). Parasite nests were also seen at this time of infection. The liver had mild inflammatory infiltrates, like those seen at 14 dpi (**Figure 4F**).

As the infection progressed, at 40 dpi, the histopathological changes were less intense in both infected mouse strains. Parasite nests were no longer observed in any organs studied and the inflammatory infiltrates observed in the different organs were milder, despite being found in the same regions described previously.

Severe areas of inflammatory infiltrate in the heart and the different cell types observed in the liver of both infected mice, lead us to question about the immune response occurred in these animals. To clarify and justify these findings, it is important to study the hematological changes and cytokines pattern produced after *T*. *cruzi* SC2005 intragastric infection, as well as characterize phenotypically the cell populations present in the inflammatory infiltrates of affected organs and circulating in the blood, in order to understand the differences in the response to infection between A and BALB/c mice.

### Hematological Analysis

**Figure 5:**
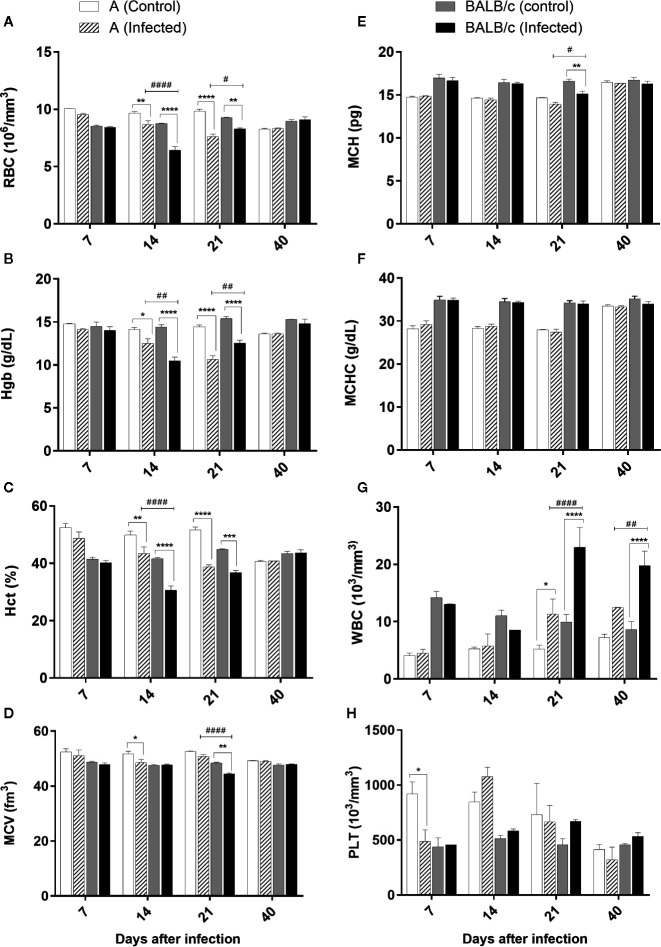
Complete blood count—Complete Blood Count (CBC) of A and BALB/c mice uninfected and intragastrically infected with 10^7^ metacyclic trypomastigote forms of *T. cruzi* SC2005 strain. **(A)** Red Blood Cell (RBC) count; **(B)** Hematocrit (Hct) levels; **(C)** Mean Corpuscular Hemoglobin (MCH); **(D)** White Blood Cell (WBC) count; **(E)** Hemoglobin (Hgb) levels; **(F)** Mean Corpuscular Volume (MCV) analysis; **(G)** Mean Corpuscular Hemoglobin Concentration (MCHC); **(H)** Platelet (PLT) count. Data represent mean ± SEM of two independent experiments of 06 animals/group/day. Statistical analyses between groups were performed using Two-way Anova and Tukey multiple comparison test. Results were considered significant with P < 0.05 (*P < 0.05; **P < 0.01; ***P < 0.001; ****P < 0.0001). * represent differences between infected and control mice, # represent differences between A and BALB/c infected mice.

CBC revealed a significant reduction in the RBC, Hgb, and Hct at 14 and 21 dpi in the infected mice of both strains, compared to uninfected mice. The decrease in the RBC, Hgb and Hct levels was significantly more pronounced in A mice at 21 dpi and in BALB/c mice at 14 dpi. After this marked decline, the parameters tended to normalize over time (**Figures 5A–C**).

A significative MCV reduction was observed in A infected mice at 14 dpi, only when compared with the control group. At 21 dpi, BALB/c infected mice showed a significant reduction in this parameter, when compared with the control group and A mice (**Figure 5D**).

Alterations in MCH were observed only at 21 dpi in BALB/c infected mice, which showed a significative reduction of this parameter when compared with the control group (**Figure 5E**).

No alterations were observed in MCHC values (**Figure 5F**).

Total white blood cell counts significantly increased in the blood of both infected lineages at 21 and 40 dpi (**Figure 5G**). BALB/c mice showed an increase significantly higher in this parameter than A mice, mainly at 21 dpi. The alteration of this parameter was characterized by an increase of the monocytes and lymphocytes, as well as the presence of a lymphocytic atypia in both infected groups.

Platelet count was significantly reduced only at 7 dpi in A infected mice when compared with the control group (**Figure 5H**).

These results showed that *T. cruzi* SC2005 infection induce an earlier hypochromic anemia (14 dpi), and a higher leukocytosis in BALB/c infected mice at 21 dpi, when compared with A infected mice. This leukocytosis was correlated with the increase of parasitemia in both infected mouse strains.

### Cytokine Quantification

**Figure 6:**
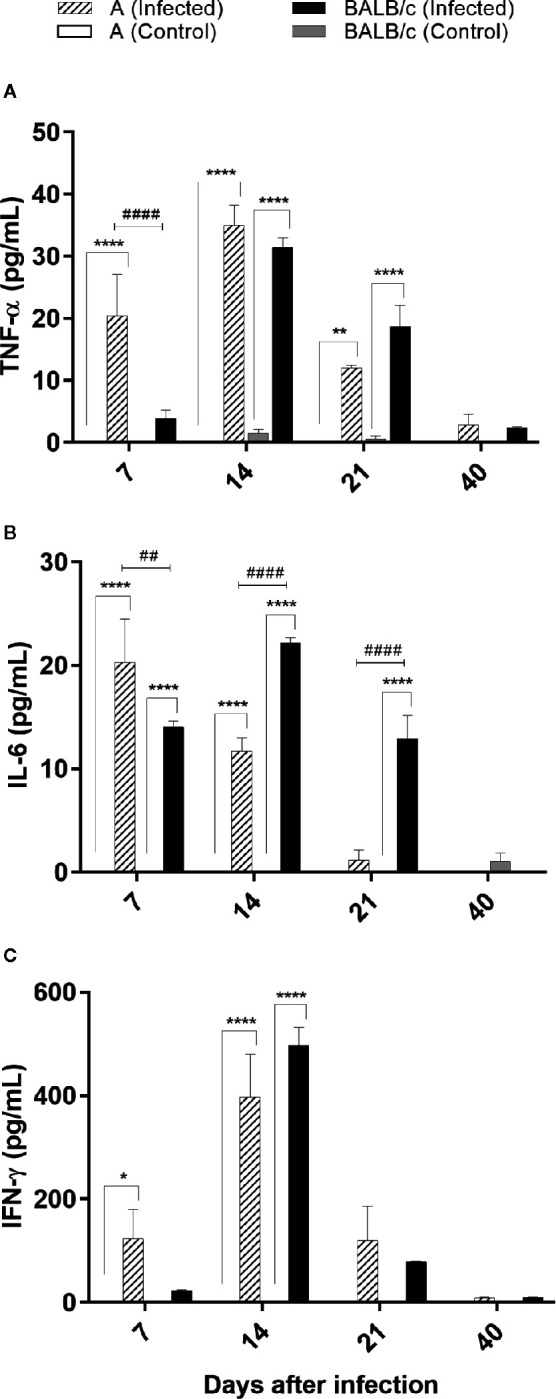
Cytokine evaluations by flow cytometry—TNF-α, IL-6 and IFN-γ levels was assessed in the serum from uninfected and *T. cruzi* SC2005 intragastrically infected A and BALB/c mice. The bars graph representing the mean ± SEM of percentages of **(A)** TNF-α; **(B)** IL-6; and **(C)** IFN-γ produced in the sera harvested from uninfected and *T. cruzi*-intragastrically-infected A and BALB/c mice in the respective time-points: 7, 14, 21, and 40 days after infection. Data represent two independent experiments with 06 animals/group/day. Statistical analyses between groups were performed using Two-way Anova and Tukey multiple comparison test. Results were considered significant with P < 0.05 (*P < 0.05; **P < 0.01; ***P < 0.001; ^****^P < 0.0001). * represent differences between infected and control mice, # represent differences between A and BALB/c infected mice.

Cytokine quantification revealed no changes in IL-10, IL-4, IL-2, and IL-17A cytokines levels. However, TNF-α, IFN-γ, and IL-6 levels were significantly increased in *T. cruzi* A and BALB/c infected mice when compared with the control groups through the infection. Monitoring of these cytokines showed an increase of TNF-α concentrations at 7, 14, and 21 dpi in A infected mice, while in BALB/c infected mice this increase was observed at 14 and 21dpi, when compared with control groups. TNF-α production was significantly different between infected groups, only at 7 dpi, being higher in A than in BALB/c mice. Both infected groups showed higher levels of TNF-α at 14 dpi (**Figure 6A**).

Increased levels of IL-6 in A mice were found at 7 and 14 dpi, with highest levels detected at 7 dpi. BALB/c infected mice showed increased levels of IL-6 at 7, 14, and 21 dpi, with highest levels at 14 dpi. IL-6 production was significantly different between infected groups, at 7, 14, and 21 dpi, being higher in BALB/c than in A mice (**Figure 6B**).

Both infected mouse strains produced a similar pattern of IFN-γ production, with a significant increase of this cytokine at 14 dpi, when compared to control groups (**Figure 6C**).

These results showed an earlier pattern of inflammatory cytokine production (7 dpi) in A infected mice, when compared with BALB/c infected mice.

### Frequencies of CD4^+^, CD8^+^, CD4^+^/CD8^+^ T, and CD19^+^B Lymphocytes and F4/80^+^ Macrophages

#### Heart

**Figure 7:**
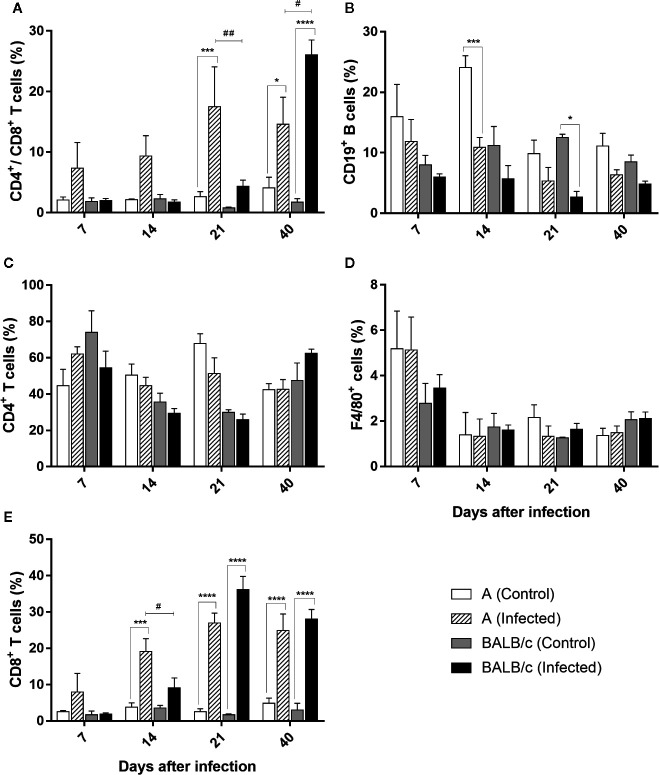
Flow Cytometry analysis of the heart - Frequencies of CD4^+^/CD8^+^ T lymphocytes **(A)**, CD19^+^B lymphocytes **(B)**, CD4^+^T lymphocytes **(C)**, F4/80+ macrophages **(D)** and T CD8^+^ T lymphocytes **(E)** in heart samples obtained from uninfected and T. cruzi SC2005 intragastrically infected A and BALB/c mice. Data represent mean ± SEM of two independent experiments with six animals/group/day. Statistical analyses between groups were performed using Two-way Anova and Tukey multiple comparison test. Results were considered significant with P < 0.05 (*P < 0.05; **P < 0.01; ***P < 0.001; ****P < 0.0001). * represent differences between infected and control mice, # represent differences between A and BALB/c infected mice.

In this organ an increased circulation of CD4^+^/CD8^+^ T lymphocytes was observed in both infected mouse strains at 21 and 40 dpi. This increase was significantly greater at 21 dpi in A infected mice (17%), when compared with control group and *T. cruzi* infected-BALB/c mice. On the other hand, in infected-BALB/c mice, this increase was significantly greater at 40 dpi, corresponding to 26% of the lymphocyte population, when compared with the control group and *T. cruzi* infected-A mice (**Figure 7A**).

The frequency of CD4^+^ T lymphocytes subpopulation in the heart from both infected mice strains showed no alteration throughout the study when compared with uninfected groups (**Figure 7B**).

The frequency of CD8^+^ T lymphocytes increase throughout the infection, showing a large increase in both infected mouse strains, when compared with control groups. In infected A mice, an increase of this subpopulation frequency was observed from 14° dpi to 40° dpi, with the largest increase at 21 dpi. The expansion of this cell type was significantly higher in this strain at 14 dpi when compared with BALB/c infected mice. The expansion of CD8^+^ T lymphocytes in BALB/c infected mice, was observed at 21 and 40 dpi, with a higher percentage of this population observed at 21 dpi (comprising 36% of the CD3^+^ T lymphocyte population) (**Figure 7C**). These results showed an earlier expansion of CD8^+^ T lymphocytes after *T. cruzi* SC2005 intragastric infection in A infected mice, when compared with BALB/c infected mice.

Quantification of CD19^+^ B cells was lower and similar throughout the experiment in both mouse strains, with exception of a reduction in the frequency of these cells in A infected mice, at 14 dpi, and in BALB/c infected mice at 21 dpi when compared to uninfected groups (**Figure 7D**).

Similar to observed in CD4^+^ T lymphocytes, the percentage of F4/80^+^ macrophages was similar to the respective control groups in both mice infected strains (**Figure 7E**).

#### Liver

**Figure 8:**
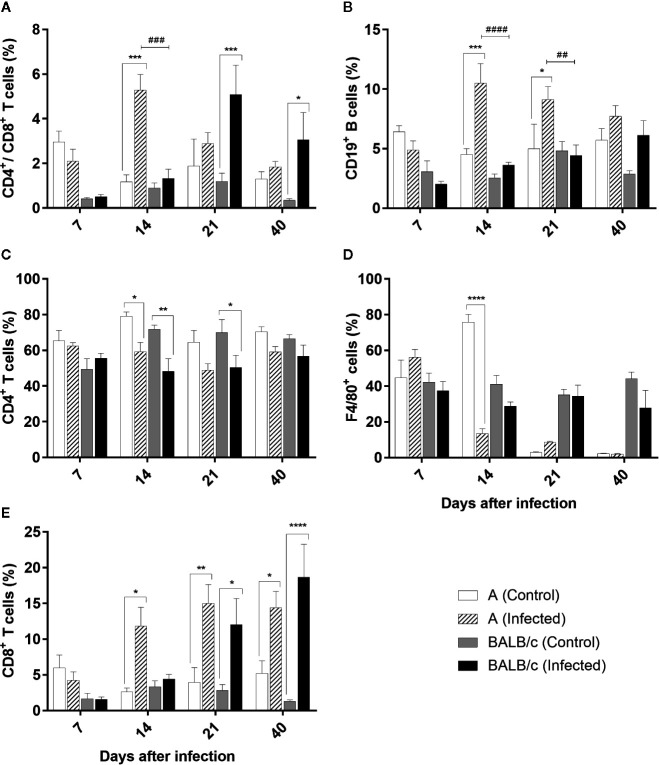
Flow Cytometry analysis of the liver - Frequencies of CD4^+^/CD8^+^ T lymphocytes **(A)**, CD19^+^B lymphocytes **(B)**, CD4^+^T lymphocytes **(C)**, F4/80^+^ macrophages **(D)** and T CD8^+^ T lymphocytes **(E)** in liver samples obtained from uninfected and T. cruzi SC2005 intragastrically infected A and BALB/c mice. Data represent mean ± SEM of two independent experiments with six animals/group/day. Statistical analyses between groups were performed using Two-way Anova and Tukey multiple comparison test. Results were considered significant with P < 0.05 (*P < 0.05; **P < 0.01; ***P < 0.001; ****P < 0.0001). * represent differences between infected and control mice, # represent differences between A and BALB/c infected mice.

Distribution profile of CD4^+^/CD8^+^ T cells in the liver showed a significative increase of these cells in A infected mice, at 14 dpi (comprising 5% of the lymphocyte population), when compared with the control group and BALB/c infected mice. On the other hand, in BALB/c infected mice this increase was observed at 21 and 40 dpi, when compared with the control groups, but not significantly different of A infected mice (**Figure 8A**).

CD4^+^ T cell subpopulations was significantly decreased in both mouse strains at 14 dpi, and at 21 dpi in BALB/c infected mice when compared with the control groups. Nevertheless, these differences were not significant between infected groups throughout all experiment in this organ (**Figure 8B**).

Contrary to observed in CD4^+^ T cells subpopulations, CD8^+^ T cells frequency increased from 14 dpi in animals infected by *T. cruzi*. An expressive increase of this cell type was observed at 14, 21, and 40 dpi in infected A animals, ranging from to 11% and 15% from CD3^+^ T lymphocyte population. In BALB/c infected mice this increase was significant at 21 and 40 dpi, reaching almost 20% at 40 dpi (**Figure 8C**).

A infected mice showed an expansion of CD19^+^ B lymphocytes at 14 and 21 dpi. The percentage of these cells was two-fold higher in the infected mice than observed in the controls and BALB/c infected mice at 14 dpi (**Figure 8D**).

The frequencies of F4/80^+^ macrophages declined significantly in A mice only at 14 dpi, when compared with control group (**Figure 8E**).

#### Blood

**Figure 9:**
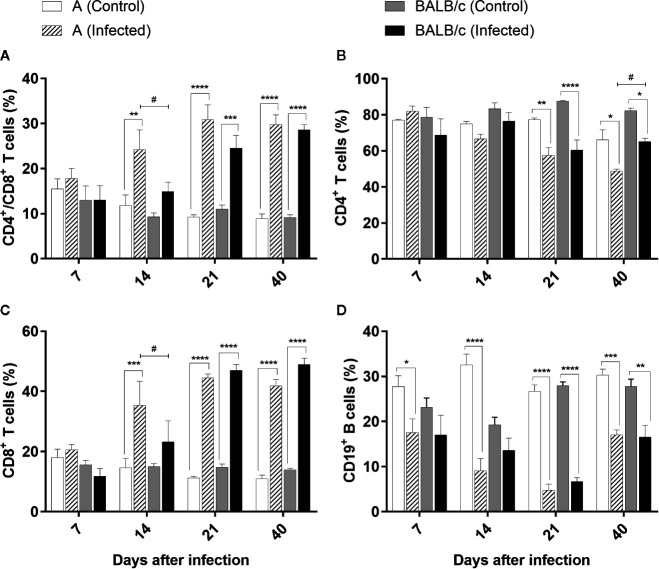
Flow Cytometry analysis of the blood - Frequencies of CD4^+^/CD8^+^ T lymphocytes **(A)**, CD4^+^T lymphocytes **(B)**, T CD8^+^ T lymphocytes **(C)**, and CD19^+^B lymphocytes **(D)** in blood samples obtained from uninfected and T. cruzi SC2005 intragastrically infected A and BALB/c mice. Data represent mean ± SEM of two independent experiments with six animals/group/day. Statistical analyses between groups were performed using Two-way Anova and Tukey multiple comparison test. Results were considered significant with P < 0.05 (*P < 0.05; **P < 0.01; ***P < 0.001; ****P < 0.0001). * represent differences between infected and control mice, # represent differences between A and BALB/c infected mice.

The evaluation of the CD4^+^/CD8^+^ T cells in the blood exhibited the same pattern in both infected groups, showing a significant difference between infected mouse strains only at 14 dpi. In A infected mice, there was a significant increase of these cells at 14, 21, and 40 dpi (ranging from 24% to 30% of the T lymphocyte population), while in the BALB/c infected mice, this increase was significant at 21 and 40 dpi (24% and 28% of the T cell, respectively) (**Figure 9A**).

CD4^+^ T lymphocyte kinetics indicated a significant reduction of this subpopulation in both infected mouse strains at 21 and 40 dpi, when compared with control groups. These reductions were significant between infected mouse strains only at 40 dpi (**Figure 9B**).

CD8^+^ T cells exhibited the same pattern observed in the CD4^+^/CD8^+^ T cells, in both infected groups, with a significant difference between infected mouse strains only at 14 dpi. CD8^+^ T cells increased from 14 dpi in A infected mice. An expressive increase of this cell type was observed at 14, 21, and 40 dpi in this group, ranging from 35% to 44% of the CD3^+^ T lymphocyte population. In the BALB/c infected mice this increase was more significant at 21 and 40 dpi, reaching almost 49% of the total of T cells (**Figure 9C**).

A reduction in the CD19^+^ B cells was observed in the blood of the animals throughout infection. A infected mice presented an expressive decrease at all the points studied. At 21 dpi the percentage of CD19^+^ B lymphocytes in the infected animals was nearly seven-fold lower than observed in uninfected mice. Likewise, BALB/c infected mice exhibited a significant reduction in this population through the infection, however, more significant at 21 and 40 dpi. At 21 dpi the reduction was more pronounced, and an average of 6.68% of CD19^+^ B cells was observed among BALB/c infected animals, while the control group had a population of around 28% (**Figure 9D**).

## Discussion

Clinical outcomes of Chagas’s disease depend on the complexity of the factors involved, related both to the parasite (genetic variability, inoculum, infectivity, pathogenicity, virulence, and inoculation pathways) and to the host (age, sex, nutrition, immune profile, and species). The interactions between these factors exert a crucial influence on the pathophysiology of Chagas disease, interfering in the host’s immune response and in the evolution of the disease (21, 27, 32, 33, 43).

The route of infection is considered an important factor for the occurrence of disease and a determinant of its evolution (22, 23). In addition to vector transmission, *T. cruzi* is primarily maintained in nature by an usual primitive character of the enzootic cycle, which the ingestion of infected vectors by several mammal species, through predatory mechanisms of survival, keeping the parasite circulation (44). Orally transmitted acute Chagas’ disease is currently the main route of transmission in northern South America and other Latin American countries (9, 44–46). Unlike the disease developed after vector-born transmission, patients infected by oral route develop a large number of signs and symptoms and present mortality rates highest (8%–35%) than patients infected by classical vectoral pathway (5%–10%) (47). These differences are probably related not only the greater efficiency of the parasite penetration in the gastric mucosa, but also usually the large quantity of parasites present in the ingested inoculum than those present in the excrement and that can penetrate through the skin (16).

In the present study, we utilized *T. cruzi* SC2005 strain (TcII), isolated from a patient during an oral outbreak of Chagas’ disease in the south region of Brazil (34), to infect inbred mice from different genetic backgrounds and compare the immunopathological alterations occurred after intragastric infection. BALB/c and A mice intragastrically infected by *T. cruzi* SC2005 showed high mortality rates, ranging from 10% to 25%, corroborating the highest mortality rates observed in patients orally infected. These mice developed a parasitemic profile similar to previously described in other studies in murine model using Swiss mice infected by *T. cruzi* strains from the same oral outbreak, showing double peaks of parasitemia, with the early low peak characteristic of biodeme type II (23, 48).

When we compare the effects of the oral infection between the two mouse strains, we observed significant differences in both parasitemia levels and percentages of survival. BALB/c mice were more susceptible to infection, showing highest parasitemia and mortality rates. Collins et al. (49) reported that the magnitude of the mucosal immune response developed after oral infection is the main factor which determines the parasite’s fate of and the level of systemic parasitemia (49). The contribution of host genetics background in pathology caused by *T. cruzi* still unsolved and studies of genetic susceptibility to Chagas’ disease are scarce. Marinho et al. (50) evaluated the pathology caused by Sylvio X10/4 *T. cruzi* in different inbred mouse strains chronically infected. C3H/HePAS mice exhibited higher cumulative mortality and pathology focused the heart, whereas A/J mice showed a mortality frequency similar to noninfected controls and liver alterations. showing that the genetic background of mice determines the development of chronic lesions (50). Since that in the present study we use the same *T. cruzi* strain to infect both mouse lineages, we also address that the differences in the parasitemia and mortality found in this study are influenced by immunological response developed by each mouse strain, which is determined by their genetic backgrounds.

Anemia is a common hematological alteration observed in infections caused by trypanosomes, being found in African trypanosomiasis (51) and in acute Chagas’ disease. A profound anemia was described by Chagas in patients infected by *T. cruzi* (52). In the present work, both infected mouse strains showed hypochromic anemia after *T. cruzi* SC2005 intragastric infection. BALB/c mice presented a more pronounced hypochromic anemia at 14 dpi, when compared with A infected mice, which showed a higher decrease in RBC and Hgb at 21 dpi. Different mouse strains infected by *T. cruzi* also showed this hematological alteration (53). Nevertheless, the mechanisms responsible for this alteration are not clearly understood yet. Anemia could be caused by an impaired maturation of bone marrow precursors (54). The reduced number of blood cells and the impaired bone marrow function are associated with the lethality rate in acute *T. cruzi* infection (55). All these alterations in the blood and bone marrow may be influenced by cytokines secretion, parasite, or cell-dependent cytotoxicity (56).

Several cytokines are related as suppressors of bone marrow activity, among them are TNF-α and IFN-γ (57–60). Binder et al. (57) have shown that the excessive production of TNF-α and IFN-γ appeared to promote damage in hematopoiesis of mice infected by lymphocytic choriomeningitis virus (LCMV). TNF-α has been also to be important as anemia mediator in studies with malaria (61). Inhibitory effects of TNF-α on erythropoiesis have been demonstrated in previous studies (60, 62), as well as during acute infection by *T. cruzi*, which the production of TNF-α by activated macrophages was correlated with a decrease in erythropoiesis (56). In our study both mouse strains infected by *T. cruzi* SC2005 produced high levels of TNF-α and IFN-γ at 14 days of infection, correlating with the decrease of RBC and Hgb. These results indicate that the anemia observed after *T. cruzi* infection can be associated with a depressed bone marrow function induced by TNF-α and IFN-γ.

Another factor that can contribute to anemia, is a reduced life span or sequestration of RBC by autoantibodies or other mechanisms, as described in other protozoan and viral infections (61, 63–67). In normal mice, the average life span of a red blood cell is 40 days (68). In the present study, BALB/c and A mice infected by *T. cruzi* SC2005 presented anemia earlier to this period, between 14 and 21 dpi, showing that *T. cruzi* infection reduces the life span of RBC contributing to anemia.

In consequence of the depressed bone marrow function, characterized by the insufficiency or poor quality of the blood elements produced, extramedullary hematopoiesis (EMH) can occur in adult mouse livers (69, 70). ECM does not occur in adult mice livers under normal physiological conditions, however, can occur under myelosuppression by various pathological lesions, including hemoglobinopathies, most commonly sickle cell anemia and thalassemia (71, 72). In our study, we observed the occurrence of extramedullary hematopoiesis, with the presence of megakaryocytes, immature hematopoietic cells and mitotic cells in the livers of *T. cruzi*-infected animals at 14 and 21 dpi. The occurrence of ECM in livers of Swiss Webster mice infected with the same *T. cruzi* strain have been previously described by us (23). Altogether, these findings corroborate the hypothesis that the anemia observed in mice infected with *T. cruzi* SC2005 is caused by an impairment in bone marrow function, induced by TNF-α and IFN-γ, which lead to occurrence of extramedullary hematopoiesis in mice livers as a compensatory mechanism.

Leukocytosis is another hematological alteration described after *T. cruzi* infection. In the present study both infected mouse strains showed a significant leukocytosis at 21 and 40 dpi, characterized by monocytosis and lymphocytosis, as well as by the presence of a lymphocytic atypia, which were associated with the parasitemia levels. Several works, using different experimental models, previously reported alterations in leukocyte counts associated with parasitemia levels. Beagle dogs infected by different *T. cruzi* strains showed a positive correlation between leukocytosis, lymphocytosis and parasitemia peaks (73, 74). The same correlation was found in cynomolgus macaque (*Macaca fascicularis*) naturally infected by *T cruzi* (75) and in Rhesus monkeys experimentally infected with *T cruzi* Colombian strain (76). Previous studies conducted by our group, also found a positive correlation between parasitemia levels and leukocytes counts in Swiss mice infected by *T. cruzi* SC2005. Contradictory results were obtained by other authors during experimental murine infection with *T. cruzi* CL strain. C3H infected-mice showed an exponential growth of parasites accompanied by leukopenia (54). Such dissimilarity in experimental data suggests that both the genetic background of experimental model and the *T. cruzi* strain may influence the impact on hematological characteristics of *T. cruzi* infection.

It is known that the increase of both Natural killer (NK) and CD8^+^ T lymphocytes is characteristic of both acute and chronic *T cruzi* infections (77). Increased levels of these cells have been described as biomarkers for Chagas disease in humans and monkeys naturally infected by *T cruzi* (78). NK cells are an important source of IFN-γ, which activates macrophages to produce nitric oxide and subsequently control the parasite growth in acute *T cruzi* infections (77–79). Moreover, the cytotoxic activity of NK cells and CD8^+^ T cells contributes to control parasite levels in the blood circulation, through the killing of parasites (78, 80, 81). As we described, BALB/c and A mice infected by *T. cruzi* SC2005 showed lymphocytosis (characterized by increase of CD8^+^ T cells) at 21 and 40 dpi. The enhancement of these T cell subpopulations occurred at the parasitemia peak and persisted until the end of the infection when there was a reduction of parasite load. It is occur probably due to cytotoxic activity of these cells, as reported previously (75).

Inflammatory infiltration was found in many organs, and lymphocytes were the most frequently observed cell types. As observed in the blood, in the heart and liver there was an increase of CD8^+^ T and CD4^+^/CD8^+^ T lymphocytes of both *T. cruzi*-infected mouse strains. CD4^+^/CD8^+^ double-positive T cells have been observed in individuals harboring infectious and autoimmune diseases, and chronic inflammatory disorders (82). Under normal conditions, these cell types are found in the thymus, where they undergo differentiation into mature CD4^+^ and CD8^+^ T cells. During *T. cruzi* infection, however, a deregulated cascade of proinflammatory cytokines significantly impaired this organ, leading to cell maturation in extrathymic organs such as the bone marrow and liver (83, 84). In the present study, the hematological findings showed a hypochromic microcytic anemia and lymphocytic atypia in infected mice during infection. This finding explains the circulation of CD4^+^/CD8^+^ T double-positive cells in the liver and blood, and indicates the maturation of the T lymphocytes expressing CD4^+^ and CD8^+^ co-receptors in the liver of A and BALB/c infected mice.

The performances of both CD4^+^ and CD8^+^ T cell subpopulations have been observed during *T. cruzi* infection, irrespective of the route of infection studied (23, 42, 49). CD8^+^ T cells have been described as being of great importance for host resistance and effective control of the parasite outgrowth during acute and chronic infections (49, 81, 85). Studies indicate that CD8^+^ T lymphocytes fail to restrain parasitemia and tissue parasitism in the absence of CD4^+^ T cells (86). This may occur because CD4^+^ deficiency leads to an increase in tissue parasitism as a consequence of the overall reduction of host immune response, possibly due to the action of CD4^+^ T lymphocytes in promoting the activation of macrophages and proliferation of CD8^+^ T and B cells (87, 88). Therefore, these results show that both CD4^+^ and CD8^+^ T subpopulations are necessary for the development of protective immunity, being CD8^+^ T cells more effective in develop an effector function against the parasite (89, 90). B cells are another cell type involved in *T. cruzi* infection. In addition to the secretion of antibodies, the role of B cells as antigen-presenting cells (APC), activating CD8^+^ T cells was described in immunization studies (91). B cells are also related to the increased mobilization capacity of the inflammatory cells to the tissues and are fundamental to trigger Th1 response that favor the control of parasite growth (77, 92, 93).

In the present study, there was an increase of both CD8^+^ T cells and CD19^+^ B cells in the hepatic parenchyma, suggesting the development of APC functioning by the CD19^+^ cells. However, A mice showed an early increase of these cells’ frequencies (14 dpi) than BALB/c infected mice (21 dpi). The liver is the main organ involved in the defense against disseminating blood pathogen, regardless of portal of entry. On this way, immune surveillance by the liver is critical to host immunity and survival (94, 95). In *T. cruzi* infection, the liver is known to be a target tissue for the parasite and exert a role in clearance of blood trypomastigotes (96). Thus, the early presence of B and CD8^+^ T cells in the liver observed in this work, indicates the importance of this organ in the parasite clearance and may explain why A mice had lower parasite load and mortality.

We also observe a decrease in the B lymphocyte frequencies in the heart and blood, and in CD4^+^ T cells in the blood and heart of infected animals. This decrease is probably caused by the migration of this cell type to other sites affected by the infection. The subsequent return to normal levels is linked to the modulation of the local and systemic immune system. This regulation is often related to the severity of the clinical manifestations present, reflecting the redistribution and circulation of lymphocyte subtypes (23, 97).

The stomach has been described as the main region of *T. cruzi* invasion in the host (98, 99). In this study, the stomach of animals from both infected lineages presented intense inflammatory infiltrates. At 14 dpi, the presence of inflammatory cells in the muscular layer of the stomach of these animals, coincided with the largest amount of parasite DNA detected. In both strains, at 21 days of infection, the stomach was still very inflamed, but the presence of parasite DNA was very low. Although some authors describe the oral cavity, esophagus and palate as possible sites of parasite invasion (100, 101), the data from the present study indicate efficient migration, invasion and multiplication of parasite in the stomach of infected animals.

The heart of the infected BALB/c and A mice also showed intense immunopathological changes. Cases of death from acute Chagas disease are closely related to cardiac damage (3, 102). BALB/c mice showed largest cardiac involvement and a higher parasite load in this organ. The extensive damage of the heart plus the large number of parasite DNA and a late CD8^+^ T cell response, corroborate to the highest number of deaths in this group.

In the liver, the presence of higher numbers of inflammatory cells around the vessels as well as in the parenchyma, indicates cellular extravasation and organ damage. In studies with BALB/c mice infected with the Tulahuén strain, the inflammatory infiltrate was not observed in the parenchyma region, but was located exclusively around the vessels (42). However, other studies describing the same findings of the present study. In independent studies using Swiss mice infected by Y or SC2005 *T. cruzi* strains, inflammatory infiltrates in parenchyma and around the liver vessels were also observed (23, 103). Sardinha et al. (96) described that the parasite and host cell interactions that occur during the initial infection by Y *T. cruzi* strain in C57BL/10 mice shows the presence of viable parasites in the liver, which are associated with the presence of focal inflammatory infiltrates in the liver parenchyma and in the perivascular spaces. These inflammatory infiltrates are composed by cells like macrophages, CD4 ^+^ and CD8 ^+^ T lymphocytes and natural killer cells, being important for parasite clearance, thus controlling parasitemia (96).

Paralleling parasitological, histological and immunophenotypical analysis, BALB/c and A infected-mice showed an enhancement of IFN-γ, TNF-α, and IL-6 Th1 cytokines in the serum, but A mice produce these cytokines earlier than BALB/c mice. These cytokines have a proinflammatory role and are involved in the parasite growth control and host resistance (104, 105). The importance of these cytokines in the response to *T. cruzi* infection has been described in several murine studies. The inhibition of IL-18 or 5-lipoxygenase in mice generated an increase in the levels of IL-12, IFN-γ, IL-1β, and IL-6, improving the resistance of mice to parasite growth during the acute phase of disease (106, 107). On the other hand, the reduced production of IFN-γ, IL-6, and TNF-α cytokines in C57BL/6 IL-17A knockout (IL-17A ^-^/^-^) mice infected with *T. cruzi* Tulahuén strain produced a more severe parasitemia and mortality, than observed in wild-type mice (108). *In vitro* studies using PBMC infected with *T. cruzi* Tulahuén strain indicate the action of IL-6 on cytotoxic cells, improving their survival and effector functions (105). These data reveal the great importance and the role of these cytokines in the control of parasite infection and mortality rates. In the present work, despite these proinflammatory cytokines were produced by both infected mice, A mice exhibited an earlier production of these cytokines, justifying the low parasitemia and mortality rate observed.

All the results point that the intragastric *T. cruzi* SC2005 infection produced a systemic response, that occur because *T. cruzi* spreads through the bloodstream, following the invasion and multiplication into the cells of different organs.

Although both mice infected strains exhibited the same profile of changes, A infected mice exhibited an early development of a cytotoxic cellular response, with a fast induction of CD8^+^ T and proinflammatory cytokines production, with lower parasite load and mortality. On the other hand, in BALB/c infected mice the response to infection occurred later, after a considerable increase in parasitemia. This later cytotoxic and proinflammatory response favored the parasite multiplication and spread, and consequent higher mortality rate in these animals.

Altogether, these results point to the host genetic background shaping the response to infection and emphasize the importance of the earlier development of a cytotoxic cellular profile, with the production of proinflammatory cytokines for a less severe manifestation of Chagas disease.

## Data Availability Statement

All datasets presented in this study are included in the article/**Supplementary Material**.

## Ethics Statement

The animal study was reviewed and approved by the Ethics Committee for Animal Research of the Fundação Oswaldo Cruz (CEUA-FIOCRUZ), license LW 42/14.

## Author Contributions

CD and KC designed the study. CD, FC, DH, AB, and MP-M collected and analyzed the data. KC acquired the funding. CD and FC were in charge of the investigation. FC, CD, DH, and MP-M were in charge of the methodology. Project administration and supervision were done by KC. CD and FC wrote the original draft of the article. CD, FC, MP-M, AB, and KC wrote, reviewed and edited the article. All authors contributed to the article and approved the submitted version.

## Funding

This work was supported by a grant from Instituto Oswaldo Cruz/Fundação Oswaldo Cruz. CD was a CAPES scholar. The funders had no role in the study design, data collection and analysis, decision to publish, or preparation of the manuscript.

## Conflict of Interest

The authors declare that the research was conducted in the absence of any commercial or financial relationships that could be construed as a potential conflict of interest.

## Supplementary Material

Supplementary Figure 1 Flow cytometry representative protocol. The gate strategy was performed, as follow: **(A)** to exclude cell aggregates from analyses, cells were gated on Singlets region in FSC-A vs. FSC-H dot-plot; **(B)** a SSC-A vs. FSC-A dot plot was created from Singlets gate and Monos region was defined; from Monos gate, **(C)** CD4+/CD3+ T lymphocytes; **(D)** CD8+/CD3+ T lymphocytes, **(E)** CD19+ B lymphocytes and **(F)** F4/80+ macrophages were determined. **(G)** CD8+/CD4+ double-positive T lymphocytes were defined from CD3+ gate in CD3 vs. FSC-A dot plot **(H)**. DN = Double Negatives.
