# Supplementary figures and images for: Host Genetics Background Influence in the Intragastric Trypanosoma cruzi Infection
Source: Front Immunol. 2020 Nov 24;11:566476. doi: 10.3389/fimmu.2020.566476 (PMC7732431; doi:10.3389/fimmu.2020.566476)

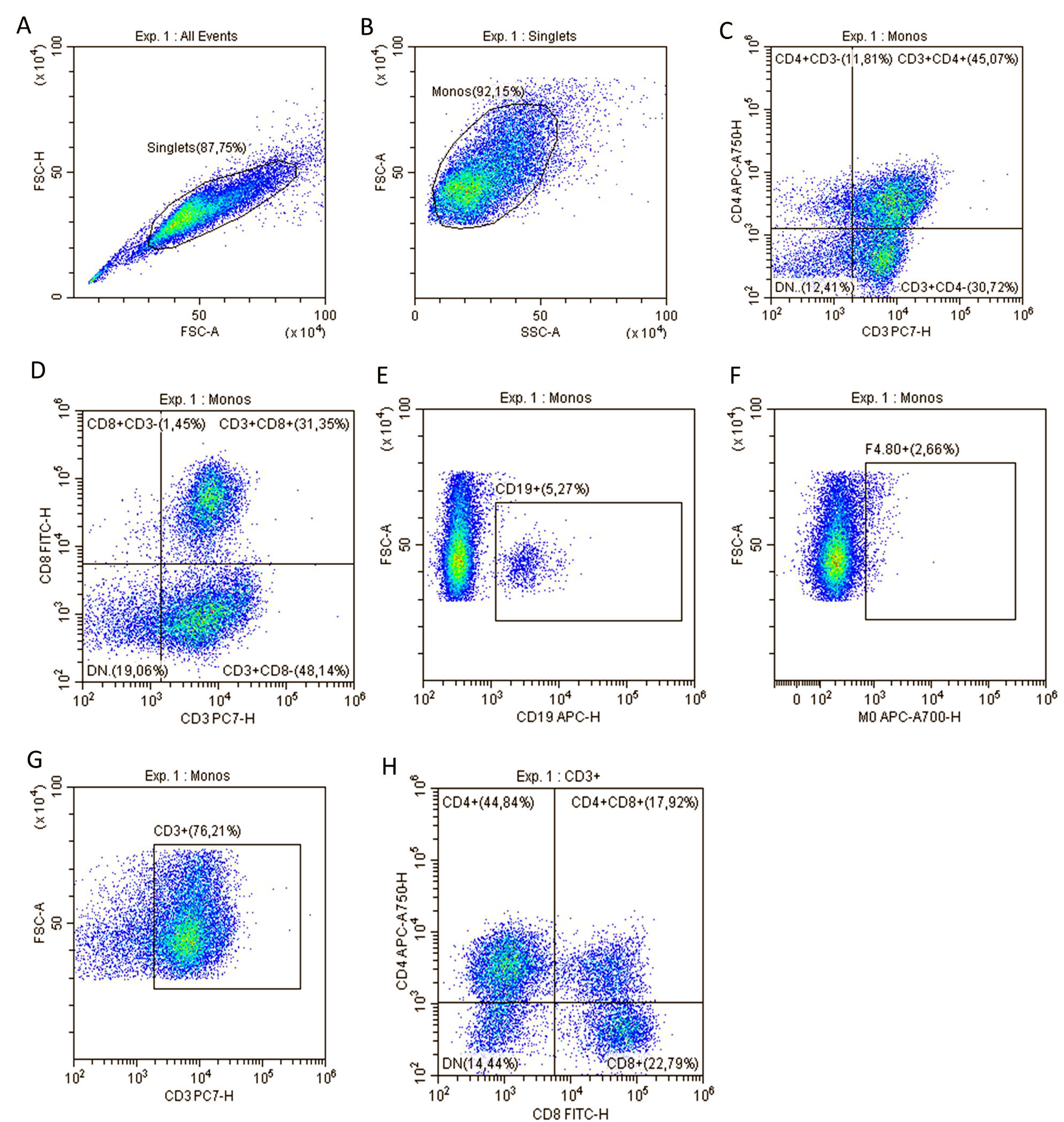

Supplement: Supplementary Figure 1 — Flow cytometry representative protocol. The gate strategy was performed, as follow: (A) to exclude cell aggregates from analyses, cells were gated on Singlets region in FSC-A vs. FSC-H dot-plot; (B) a SSC-A vs. FSC-A dot plot was created from Singlets gate and Monos region was defined; from Monos gate, (C) CD4+/CD3+ T lymphocytes; (D) CD8+/CD3+ T lymphocytes, (E) CD19+ B lymphocytes and (F) F4/80+ macrophages were determined. (G) CD8+/CD4+ double-positive T lymphocytes were defined from CD3+ gate in CD3 vs. FSC-A dot plot (H). DN = Double Negatives. [file Image_1.tif]
